# Decorated‐Induced Oxygen Vacancy Engineering for Ultra‐Low Concentration Nonanal Sensing: A Case Study of La‐Decorated Bi_2_O_2_CO_3_

**DOI:** 10.1002/advs.202408096

**Published:** 2024-09-28

**Authors:** Zichen Zheng, Kewei Liu, Yiwen Zhou, Kaichun Xu, Yifan Luo, Jiabao Ding, Carla Bittencourt, Marc Debliquy, Chao Zhang

**Affiliations:** ^1^ College of Mechanical Engineering Yangzhou University Yangzhou 225127 P. R. China; ^2^ Research Institute for Materials Science and Engineering, Chimie des Interactions Plasma‐Surface University of Mons 20 Place du Parc Mons 7000 Belgium; ^3^ Service de Science des Matériaux, Faculté Polytechnique University of Mons Mons 7000 Belgium

**Keywords:** bismuth subcarbonate, decoration, gas sensor, oxygen vacancy, room temperature

## Abstract

La‐decorated Bi_2_O_2_CO_3_ (BCO‐La) microspheres are synthesized using a facile wet chemical strategy for sensing low‐concentration nonanal (C_9_H_18_O) at room temperature. These BCO‐La gas sensors are applied to evaluate agricultural product quality, specifically for cooked rice. The sensitivity of the BCO‐6La sensor significantly surpassed that of the pure BCO sensor, achieving a response value of 174.6 when detecting 30 ppm nonanal gas. Notably, the BCO‐6La sensor demonstrated a faster response time (36 s) when exposed to 18 ppm of nonanal. Additionally, the selectivity toward nonanal gas detection is higher (approximately 4–24 times) compared to interfering gases (1‐octanol, geranyl acetone, linalool, hexanal, 2‐pentyfuran, and 1‐octen‐3‐ol) during cooked rice quality detection. The gas sensing mechanism and the factors contributing to the enhanced sensing performance of the BCO‐La microspheres are demonstrated through in situ FT‐IR spectra and DFT analysis while the realistic detection scenario is carried out. In a broader context, the reported sensors here represent a novel platform for the detection and monitoring of gases released by agricultural products during storage.

## Graphical abstract

Semiconductor chemiresistive gas sensors using La‐Decorated Bi_2_O_2_CO_3_ microspheres attain high response (Ra/Rg = 174.6 @ 30 ppm), good selectivity, reproductivity, and long‐term sensing at room temperature, which provides a practical approach to design and fabricate high precision gas sensors working at room temperature.

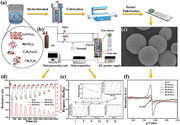

## 1 Introduction

Nonanal (C_9_H_18_O), a volatile organic compound (VOC), is primarily produced through the reduction of the carboxyl group in nonanoic acid (C_9_H_18_O_2_). It has been identified as a metabolite in cancer cell metabolism and serves as a biomarker in breath analysis for COVID‐19.^[^ ^1^, ^2^ ^]^ Notably, the concentration of nonanal in exhaled breath of lung cancer patients is significantly elevated compared to that of healthy individuals.^[^ ^3^, ^4^ ^]^ The Cancer Odor Database also indicates that nonanal levels are markedly higher in the biological samples from patients with colorectal, breast, ovarian, gastric, and esophageal cancers relative to healthy controls.^[^ ^5^ ^]^ Additionally, nonanal has been reported to play a crucial role in influencing the flavor characteristics of cooked rice and other agricultural products.^[^ ^6^, ^7^, ^8^ ^]^ A subtle aroma in rice is associated with low aldehyde concentrations, whereas elevated levels contribute to a pronounced, fatty flavor.^[^ ^9^ ^]^ Consequently, analyzing the VOCs released during cooked rice storage can provide valuable insights into its flavor characteristics, facilitating the optimization of cooking techniques and storage conditions. Extensive research has been conducted to detect nonanal. **Table** **1** provides a comprehensive summary of the reported results regarding sensing properties. Despite these advancements, numerous sensing methodologies still struggle with significant challenges, particularly concerning low sensitivity. This persistent limitation highlights the need for innovative approaches to enhance detection capabilities and improve the reliability of nonanal sensing in various applications.

**Table 1:** Recent reports on sensing properties of nonanal detection.

Materials	Working temperature [°C]	Concentration [ppm]	Response [Ra/Rg]	Response time [s]	Ref.
Ru‐loaded W_18_O_49_	Room temperature	30	16.1	25	[10]
SnO_2_ nanosheet	300	1	1.02	23	[11]
ZnO nanowire	200	2.48	4.3	323	[12]
Pt, Pd, Au/SnO_2_	300	9.5	2.45	24	[13]
SnO_2_ particulate film	250	10	12	109	[14]
Sb_2_WO_6_ (pH 4)	Room temperature	9	7.44	32	[15]
La‐decorated Bi_2_O_2_CO_3_	Room temperature	18	86.7	36	This work

Ra and Rg refer to the resistance of the sensor in ambient air and the target analyte, respectively.

Chemiresistive gas sensors (CGSs) have garnered considerable attention across various fields due to their cost‐effectiveness, ease of miniaturization, and environmental sustainability. These attributes render CGSs particularly valuable for applications in public security, environmental monitoring, and healthcare diagnostics.^[^ ^16^, ^17^, ^18^, ^19^ ^]^ CGSs based on metal oxide semiconductors (MOS‐based CGSs) have been widely applied due to their high sensitivity, low cost, ease of use, and long‐term stability. Specifically, MOS‐based CGSs are broadly used and known for their high sensitivity, affordability, user‐friendliness, and long‐term stability. Numerous studies have documented the performance of binary MOS‐based CGSs in gas detection, demonstrating both high sensitivity and low detection limits. However, a significant limitation of these sensors is their requirement for elevated operating temperatures, typically exceeding 250 °C, which constrains their practical applications.^[^ ^20^ ^]^ To address this limitation, our research focuses on bismuth subcarbonate (Bi_2_O_2_CO_3_), a ternary MOS, as the active layer material for the detection of nonanal at room temperature. Bi_2_O_2_CO_3_ (BCO) is classified as an n‐type MOS with a structure related to the Aurivillius/Sillén phases, and it has attracted considerable interest as a functional semiconductor material. Huang et al. synthesized BCO nanosheets coated with In(OH)_3_·xH_2_O nanocomposite through a two‐step hydrothermal methodology for the engineering of sensing layers for real‐time isopropanol detection. These sensors exhibited an extraordinary response value of 20.39 at 100 ppm, with detection capabilities spanning concentrations from 1 to 1000 ppm, operating at a reduced temperature of 100 °C.^[^ ^21^ ^]^ To enhance the photocatalytic stability of oxygen vacancies (O_V_) in BCO, Chen et al. developed an effective method to prepare Bi‐metal nanoparticle‐modified BCO nanosheets, showing high photocatalytic activity and stability. The synergistic interaction between Bi‐metal nanoparticles and induced O_V_ suppresses the formation of poisonous intermediate (NO_2_), facilitating the conversion to the final product, NO_3_^−^.^[^ ^22^ ^]^ Zai et al. reported that I^−^ can partially substitute CO_3_^2−^ in BCO, resulting in a reduced bandgap and increased visible light absorption, leading to the synthesis of rose‐like I‐doped BCO microstructures via a hydrothermal strategy. Photoelectrochemical testing confirmed that I^−^ doping reduces the bandgap by introducing intermediate energy levels within the bandgap. Optimized I‐doped microspheres exhibited superior photocatalytic performance, degrading Rhodamine B in 6 min and achieving ≈90% reduction of Cr(VI) in 25 min under visible light (λ > 400 nm).^[^ ^23^ ^]^ Notably, materials based on BCO have demonstrated exceptional catalytic performance. Given the similarity between catalytic reactions and gas‐sensing mechanisms, which involve the chemical interaction of target molecules with the sensor active layer material, BCO presents substantial potential for diverse gas‐sensing technology. The structural characteristics of BCO, comprising alternating layers of [Bi_2_O_2_]^2+^ and CO_3_^2−^, facilitate the formation of 2D nanosheet structures.^[^ ^24^, ^25^ ^]^ However, these densely packed nanosheet structures may not provide sufficient active sites for effective interactions between active layer surface and target molecules. Consequently, it is imperative to explore the engineering of BCO‐based materials featuring porous 3D structures enhanced through the addition of transition metal atoms, to improve their functional performance.

In chemical sensors, the gas‐sensing process involves the catalysis of gas molecules, with the surface chemical composition, crystalographic configuration, and morphology of sensing materials significantly influencing catalytic reactions and gas‐sensing efficiency. Among various strategies to increase the performance of catalytic materials, the incorporation of transition metal atoms serves as a method to optimize grain size, create O_V,_ and modify surface states, thereby enhancing the interaction between the surface of materials and target molecules and, consequently, the catalytic activity. In particular, lanthanide (La) has been identified as an efficient catalyst for achieving the requisite selectivity and sensibility for practical applications, in addition to reported improvements in the physicochemical properties of MOSs.^[^ ^26^, ^27^, ^28^ ^]^ Gao et al. synthesized La‐doped cadmium tin oxide microcubes through coprecipitation and annealing processes exhibiting excellent ethanol gas sensing properties. The La@CdSnO_3_ sensor exhibited a response value of 115.2–100 ppm ethanol, ≈19 times higher than that of pure CdSnO_3_ at an optimal working temperature of 300 °C, alongside rapid response‐recovery rates and good stability.^[^ ^29^ ^]^ Shingange et al. investigated the H_2_S sensing potential of ZnO nanofibers (NFs) synthesized through electrospinning, followed by La‐doping and post‐calcination treatments at different doping concentrations. Their comparative analysis demonstrated that La‐doped ZnO NF‐based sensors exhibited enhanced response and faster response/recovery times while exhibiting high selectivity toward H_2_S.^[^ ^30^ ^]^ Additionally, Wang et al. reported that carboxylated graphene oxide decorated with La particles displayed enhanced antibacterial properties.^[^ ^31^ ^]^ Furthermore, Yuksel et al. investigated H_2_ adsorption and sensing on La‐doped/decorated carbon nanotube and graphene structures, revealing that La modification significantly enhances H_2_ interactions in both materials.^[^ ^32^ ^]^

In this work, we report a novel one‐step hydrothermal method for the synthesis of La‐decorated BCO microspheres, achieved through control of reaction kinetics involving urea as a stabilizer and trisodium citrate as a precipitant. The resulting material, optimized for nonanal detection, exhibited a spherical morphology with an average diameter of 3.5 µm and a highly permeable lamellar structure composed of 2D nanosheets. The gas‐sensing tests demonstrated that sensors with 6 at% La‐decorated BCO active layers exhibited exceptional detection capabilities for nonanal, achieving a sensitivity of 174.6 toward 30 ppm of nonanal at room temperature, a rapid response time of 36 s at 18 ppm and good long‐term stability. The underlying sensing mechanism was identified by in situ Fourier transform infrared (FT‐IR) spectra, revealing that nonanal is initially converted to intermediate gas species that subsequently decompose into CO_2_ and H_2_O. Density functional theory (DFT) calculation indicated that the adsorption energy of the active layer surface increased after La decoration, suggesting enhanced adsorption strength and stability, thereby contributing to enhanced gas sensing performance. Therefore, the rapid response and sensitivity enhancement associated with the La decoration are instrumental in advancing practical applications of nanomaterials‐based gas sensors for high‐performance online agricultural product inspection.

## 2 Experimental Section

### 2.1 Chemical Reagents

All the reagents employed in this study were of analytical grade and used without further purification. The following chemicals were purchased from Aladdin Biochemical Technology Co., Ltd., China: Bismuth nitrate pentahydrate (Bi(NO_3_)_3_·5H_2_O) (AR grade, CAS: 10035‐06‐0), lanthanum nitrate hexahydrate (La(NO_3_)_3_·6H_2_O) (AR grade, CAS: 10277‐43‐7), urea (CH_4_N_2_O) (AR grade, CAS: 57‐13‐6), trisodium citrate dihydrate (C_6_H_5_Na_3_O_7_·2H_2_O) (AR grade, CAS: 6132‐04‐3), and nonanal (C_9_H_18_O) (AR grade, CAS: 124‐19‐6).

### 2.2 Synthesis of Hierarchical BCO Microspheres

The synthesis of BCO microspheres was performed following the procedure reported by Huang et al.^[^ ^33^ ^]^ Initially, 0.97 g of Bi(NO_3_)_3_·5H_2_O and varying quantities of La(NO_3_)_3_·6H_2_O (0, 0.018, 0.036, 0.052, 0.070, and 0.866 g) were individually and uniformly dispersed in 70 mL of deionized water in separate containers. Subsequently, 0.30 g of CH_4_N_2_O and 1 g of C_6_H_5_Na_3_O_7_·2H_2_O were added to each solution. The ratio of La with respect to Bi was adjusted to obtain x% (La/Bi = 2 at%, 4 at%, 6 at%, 8 at%, and 10 at%). Following sonication for 5 min and magnetic stirring for 60 min to ensure homogeneity, the resulting solution was transferred to a 100 mL Teflon‐lined stainless‐steel autoclave and heated at 180 °C for 20 h. After undergoing six washes through centrifugation with ethanol and deionized water, the samples were collected and dried at 70 °C. The obtained precursor was then subsequently calcined at 300 °C for 30 min under atmospheric conditions, with a heating rate of 2 °C per minute. The resulting products, including pure BCO and La‐decorating BCO variants, were labeled as BCO, BCO‐2La, BCO‐4La, BCO‐6La, BCO‐8La, and BCO‐10La, respectively.

### 2.3 Characterization

The crystallographic structure of the samples was examined using X‐ray diffraction (XRD) on a Bruker D8 Advance instrument. Morphological characteristics were evaluated through field emission scanning electron microscope (FE‐SEM, model S4800) and high‐resolution transmission electron microscopy (HRTEM, Tecnai model). FT‐IR spectroscopy was performed on an Agilent 660‐IR spectrometer utilizing the KBr pellet technique for infrared spectra acquisition. UV‐visible absorption spectra and determination of bandgaps were performed using a UV–vis–NIR spectrophotometer (Cary 5000, Varian, USA). The chemical states of the atoms and elemental relative concentration were investigated by X‐ray photoelectron spectroscopy (XPS, Thermo Fisher ESCALAB250Xi). A high‐angle annular dark‐field scanning transmission electron microscope (HAADF‐STEM) with energy dispersive spectroscopy (EDS) was used to determine the elemental spatial distribution. The energy calibration of the XPS spectra used the C 1s peak at 284.8 eV. Surface O_V_ were identified through electron paramagnetic resonance (EPR) with a Bruker A300‐10/12 instrument. The Brunauer–Emmett–Teller (BET) surface area and pore size distribution were determined using a specific surface and pore size analyzer (Autosorb IQ3, Quantachrome Instruments). The metal content in the samples was determined by inductively coupled plasma mass spectrometry (ICP‐MS, Optima 7300 DV, PerkinElmer, USA) and a flame atomic absorption spectrophotometer (FLAA, PinAAcle 900F model, PerkinElmer, USA). The In situ FT‐IR spectra were recorded using a Bruker INVENIO S infrared spectrometer with 0.4 cm^−1^ resolution which was equipped with DTGS detector and liquid nitrogen‐cooled MCT detector.

### 2.4 Gas Sensor Platform

**Figure 1:**
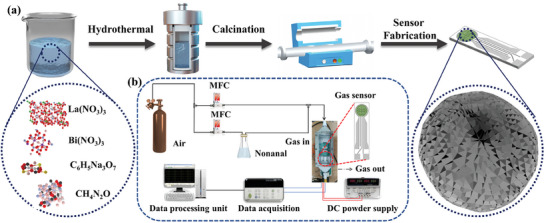
a) Schematic illustration for fabricating La‐decorating BCO microspheres and b) the setup for gas sensing tests.

The sensor electrode was fabricated using an alumina substrate with dimensions of 6 by 30 mm. The substrate was screen‐printed with platinum paste to form a heater and measurement electrodes. A 0.42 mm gap was maintained between the electrodes for gear shaping. Prior to the deposition of the sensing film, the sensor substrates underwent a cleaning procedure involving sonication in deionized water and absolute alcohol. The gas sensing measurement setup, comprising four channels for resistance measurement incorporated programmable temperature control. The coated sensors were preheated for ≈10 h at 200 °C and subsequently conditioned in preparation for gas sensing tests at room temperature. This conditioning process involved the flow of high‐purity air (comprising 79% N_2_ and 21% O_2_) for 3–5 h to ensure a stable resistance baseline before starting the actual tests. For measuring the nonanal concentration, a setup was conceived, as shown in **Figure** **1**b. The vapor pressure of nonanal was 0.26 mmHg at room temperature. The VOC concentration was calculated through Equation (1).^[^ ^34^ ^]^ Standard nonanal gas at a concentration of 342 ppm (Shanghai Aladdin Biochemical Technology Co., Ltd.) was regulated using a mass flow controller (MFC) alongside another MFC controlling the entry of high‐purity mixed air (Nanjing Special Gas Factory Co., Ltd.).

(1) $$\begin{array}{ccc} & & \mathrm{Concentration}\;\mathrm{of}\;\mathrm{VOCs}\;(\mathrm{ppm})=\\ & & \left(\mathrm{vapor}\;\mathrm{pressure}\;\mathrm{of}\;\mathrm{VOCs}\left(\mathrm{mmHg}\right)/760\right)\times 10^{6} \end{array}$$

Data was acquired through a desktop computer equipped with an analog‐to‐digital (A/D) converter and a laboratory DC power supply (GPS‐3303C) to measure the electrical resistance across the channels. The nonanal concentration was determined by independently adjusting the flow rates of nonanal and air, using the formula (1000x)/(x+y) ppm. All tests were conducted at room temperature (25 ± 2 °C), with the average relative humidity maintained at approximately 15–20%. The concentration of VOC in ppm and additional details regarding the gas sensor platform can be found in the previous studies.^[^ ^15^, ^35^ ^]^

## 3 Results and Discussion

### 3.1 Morphology and Structural Characterization

**Figure 2:**
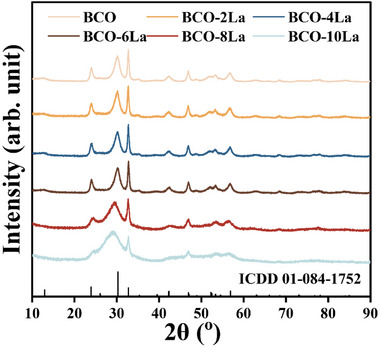
XRD patterns of all samples.

XRD analysis was used to determine the crystal structures of the samples. As depicted in **Figure** **2**a, the XRD patterns of the samples exhibited a high degree of correlation with the orthorhombic BCO phase (JCPDS Card No. 84–1752),^[^ ^36^ ^]^ thereby confirming their high purity, consistent with TEM results. With an increase in the level of La decoration, the characteristic peaks gradually broaden, particularly in BCO‐8La and BCO‐10La samples, indicating a reduction in crystalline quality and a tendency toward atomic disorder. To further comprehend the crystal structure of the samples and its correlation with gas‐sensing performance, we calculated the crystallite sizes for all samples, as detailed in Tables S1–S6 (Supporting Information). The average crystallite sizes were determined to be 10.15 nm for BCO, 9.96 nm for BCO‐2La, 10.78 nm for BCO‐4La, 10.94 nm for BCO‐6La, 7.69 nm for BCO‐8La, and 4.57 nm for BCO‐10La, with BCO‐6La exhibiting the largest crystallite size. Given its superior response to nonanal, it can be suggested that the increased crystallite size facilitates the continuity of the conductive pathways, thereby enhancing sensitivity and conferring improved stability and selectivity.

**Figure 3:**
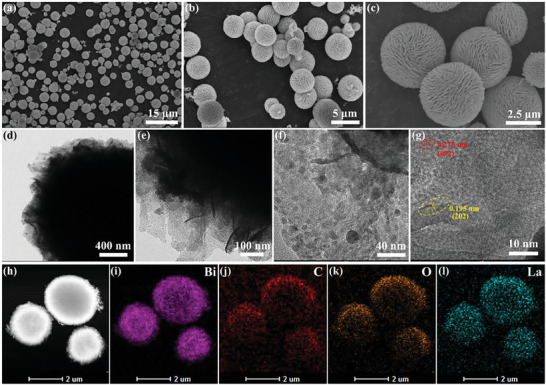
a–c) FESEM image of BCO‐6La, d–g) high‐resolution TEM images of BCO‐6La, h–l) EDS elemental mapping results of BCO‐6La.

The FESEM images of BCO‐6La, a representative sample, reveal the presence of non‐aggregated spherical particles (**Figure** **3**a), indicating a homogeneous dispersion of particles without agglomeration. Statistical analysis indicates an average diameter of 3.49 µm, as shown in Figure S2 (Supporting Information). Detailed SEM images at higher magnification reveal that each microsphere exhibits a highly permeable, layered microstructure consisting of loosely stacked 2D nanosheets (Figure 3b,c). HRTEM and high‐resolution SEM characterization confirm that the loose stacking of nanosheets (with a spacing of ≈150 nm) within individual microspheres forms a lamellar structure, enhancing the permeability of BCO‐6La (Figure 3d; Figure S3, Supporting Information). As shown in Figure 3e,f, some evenly distributed La nanoparticles can be observed on the surface of bismuth subcarbonate. Additionally, the examination of BCO decorated with varying La concentrations (2‐10 at%) indicates that La decorating did not significantly alter the layered structure (Figure S1, Supporting Information). Distinct lattice fringes were observed for BCO‐6La, with lattice spacings of 0.270 nm and 0.195 nm corresponding to the (002) and (202) planes of BCO, respectively (Figure 3g). HAADF and EDS mapping of BCO‐6La demonstrate a uniform distribution of Bi, C, O, and La, confirming effective decorating.

**Figure 4:**
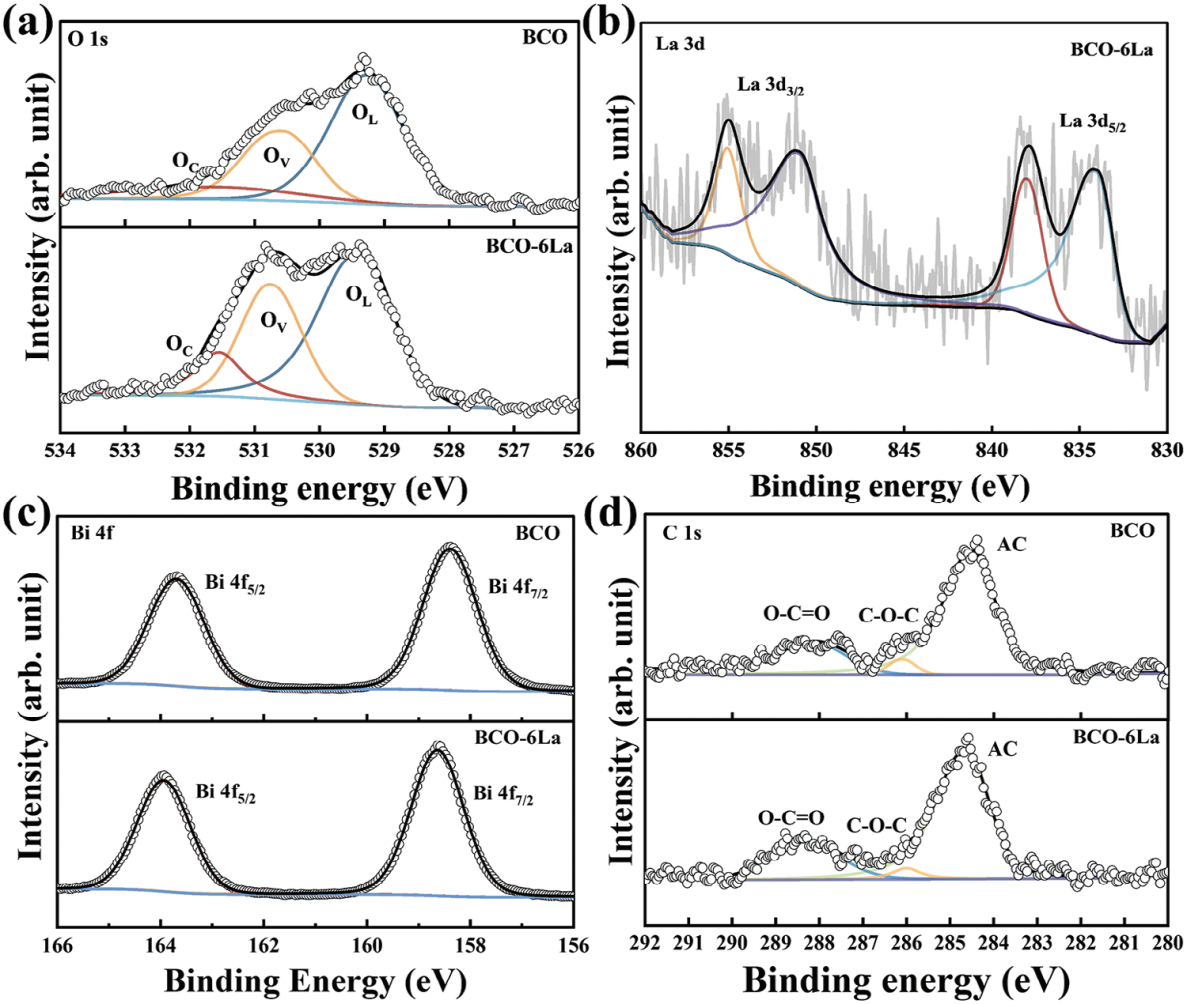
High‐resolution XPS spectrum of BCO and BCO‐6La for a) O 1s, b) La 3d, c) Bi 4f, and d) C 1s.

XPS analysis was used to determine the chemical states of elements present in the sample. The XPS spectra of BCO‐6La (Figure S5, Supporting Information) exhibited peaks corresponding to Bi 4f, C 1s, O 1s, and La 3d, indicating the presence of these elements, in agreement with EDS analysis. The fitting analysis of the O 1s spectra in **Figure** **4**a exhibits three components attributed to lattice oxygen (O_L_), O_V_, and chemisorbed oxygen (O_C_). Specifically, O_L_ refers to oxygen atoms that occupy lattice positions within the crystal structure of solid materials, while O_V_ refers to oxygen vacancies, which are characterized by the absence or displacement of oxygen atoms from their positions within the crystal structure. This defect manifests as the lack of one or more oxygen atoms within the lattice. The presence of oxygen vacancies may lead to a lower binding energy peak due to the reduced oxidation state of surrounding metal ions. O_C_ denotes molecular oxygen or oxygen atoms that are adsorbed onto the surface or interface of a material.^[^ ^37^, ^38^, ^39^ ^]^ For BCO‐6La, these components are centered at 529.3, 530.7, and 531.5 eV, respectively, while for BCO, the corresponding values are 529.3, 530.6, and 531.6 eV, respectively. From this analysis, the amount of O_V_ in BCO‐6La was 32.4%, higher than that in the BCO sample (29.3%). The higher O_V_ content can be linked to enhanced gas sensitivity.^[^ ^40^, ^41^ ^]^ Furthermore, in Figure 4b, the La 3d spectrum of BCO‐6La exhibits the spin‐orbit components (3d_3/2_ and 3d _5/2_) accompanied by their multiplet splitting, with the La 3d_5/2_ peak at 834.2 eV indicating the presence of La in La─O bonds, suggesting the successful decorating of BCO with La^3+^ ions.^[^ ^29^, ^42^ ^]^ Figure 4c presents the high‐resolution Bi 4f spectra for both BCO and BCO‐6La samples. The components of the Bi 4f doublet, in the BCO spectrum, are located at 158.4 eV (Bi 4f_7/2_) and 163.7 eV (Bi 4f_5/2_). These components are shifted 0.2 eV toward high binding energy in the BCO‐6La spectrum, indicating the successful La decorating in the BCO lattice.^[^ ^43^ ^]^ The C 1s spectrum of BCO‐6La (Figure 4d) was fitted with three components. The one centered at 284.6 eV was attributed to the presence of adventitious carbon (AC) species, while the component at 286.0 eV originated from ether groups and at 288.3 eV from ester groups, consistent with the C 1s recorded on the BCO sample.^[^ ^44^, ^45^ ^]^

**Figure 5:**
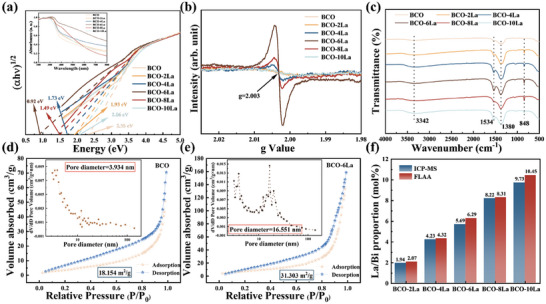
a) The UV–vis absorption spectrum in the inset with the corresponding Tauc curve, b) EPR spectra, and c) the FT‐IR spectroscopy of all samples, nitrogen sorption isotherms, and pore size distributions (inset) of the d) BCO and e) BCO‐6La. The pore size distributions are determined by the BJH model based on the absorption branches, f) The La/Bi proportion of all samples analyzed by ICP‐MS and FLAA.

UV‐Vis analysis demonstrates that La decorating enhances light absorption within the 500–675 nm range (see **Figure** **5**a inset). The bandgap of the samples was determined to be 2.35 eV for BCO, 1.93 eV for BCO‐2La, 1.73 eV for BCO‐4La, 0.92 eV for BCO‐6La, 1.49 eV for BCO‐8La, and 2.06 eV for BCO‐10La.^[^ ^23^ ^]^ Figure 5a indicates that La‐decorating has a significant impact on UV‐visible absorption characteristics. Therefore, the improved sensing performance of BCO‐6La is primarily due to its high concentration of O_V_ (XPS analysis) and narrow bandgap, facilitating oxygen adsorption and carrier transfer. During the synthesis, decorating La particles on the surface of BCO can enhance the O_V_ in the material, mainly because of the promotion of oxygen diffusion and migration. The electronic structure and chemical environment at the interface between La metal particles and BCO can reduce the diffusion energy barrier of oxygen ions, making it easier for oxygen ions to escape from the lattice, thereby forming O_V_. Furthermore, the presence of La metal particles can introduce local stress or generate local structure distortions on the surface of BCO. Such stress and distortions can change the lattice energy of the material, reducing the formation energy of O_V_, thus making it easier to generate O_V_. The abundance of O_V_ in BCO‐6La promotes charge spacing and electron depletion layer formation, enhancing sensing capabilities.^[^ ^46^, ^47^, ^48^ ^]^

EPR spectroscopy was used to evaluate the O_V_. As illustrated in Figure 5b, BCO‐6La exhibits a distinct peak (g = 2.003), significantly more intense than that of BCO, suggesting that La decorating induces more O_V_ in BCO microspheres which is in agreement with XPS results.^[^ ^29^ ^]^ FT‐IR measurement (Figure 3c) shows absorption bands at 848 cm^−1^ corresponding to CO_3_^2−^ bending modes. Bands at 1380 and 1534 cm^−1^ are associated with CO_3_^2−^ antisymmetric vibration modes.^[^ ^49^ ^]^ Additionally, the broad absorption bands at ≈3342 cm^−1^ are assigned to O─H vibrations.^[^ ^50^ ^]^ These findings, supported by XRD, XPS, EPR, and FT‐IR analyses, confirm the successful synthesis and La‐decorating of BCO.

The N_2_ adsorption‐desorption isotherms observed for BCO and BCO‐6La in Figure 5d,e fall under type IV‐H3, indicating the synthesis of mesoporous materials characterized by slit pore structures resulting from the stacking of particles. Calculations demonstrate specific surface areas of 18.154 m^2^/g for BCO and 31.303 m^2^/g for BCO‐6La. A larger specific surface area provides more active sites, which enhances the gas‐sensing properties of materials.^[^ ^12^ ^]^ Additionally, analyses using ICP‐MS and FLAA were conducted to investigate the metal elements within all samples, as shown in Figure 5f. These analyses revealed that the ratio between La and Bi content aligns with the initial experimental design, providing further evidence of the successful incorporation of La.

### 3.2 Sensing Properties

**Figure 6:**
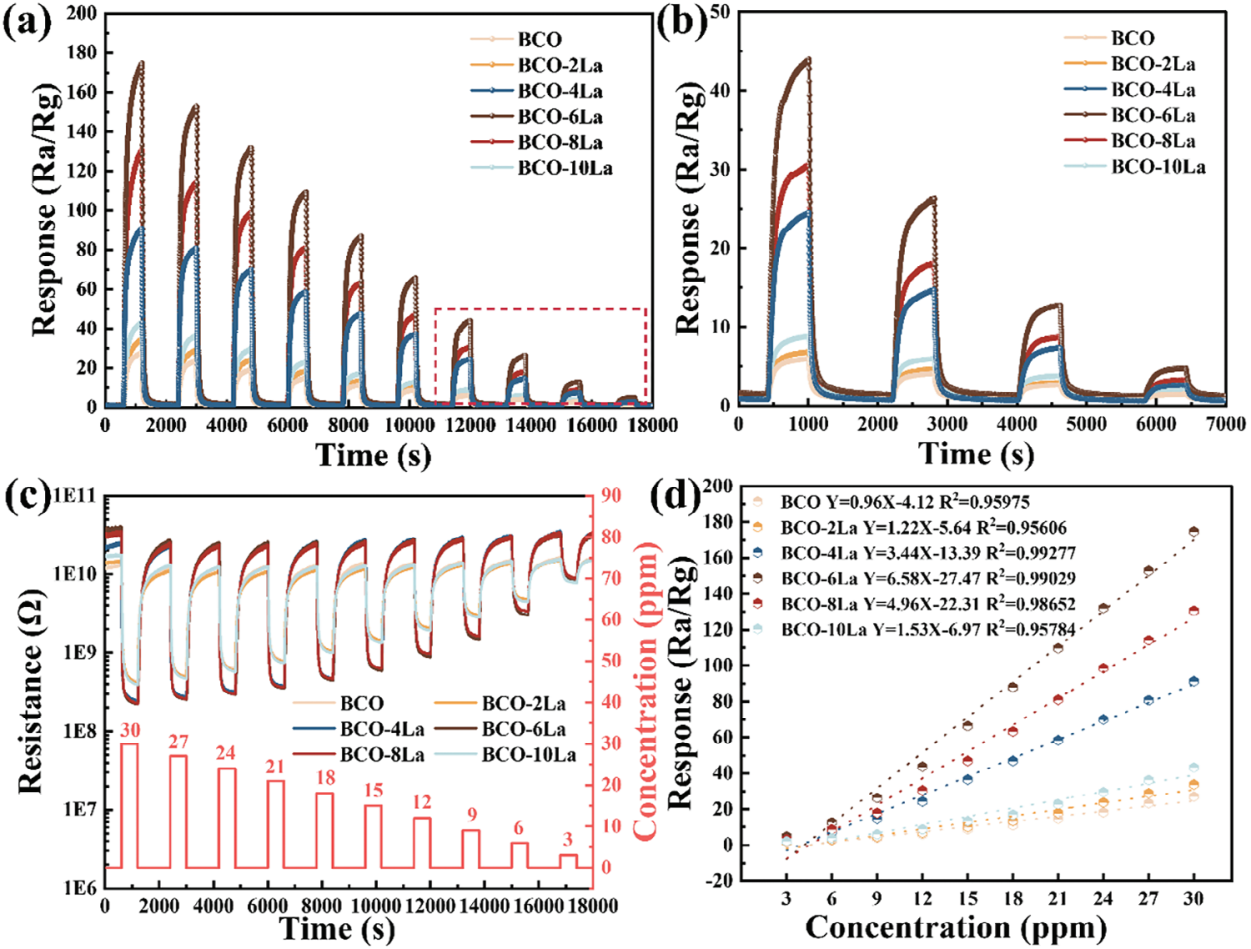
a) Transient response BCO‐xLa film to nonanal concentrations of 3–30 ppm at room temperature (25 ± 2 °C), b) magnified area of Figure 6a,c) resistive response of BCO‐xLa gas sensors toward exposure to 3–30 ppm of nonanal at room temperature (25 ± 2 °C), d) linear relationship between response verse nonanal vapor concentrations.

In this section, the performance of La‐decorated BCO microspheres in nonanal sensing was evaluated. The sensor response value was defined as Ra/Rg while the definition of response and recovery time were consistent with the study of Jeong et al.^[^ ^16^ ^]^ The transient curves illustrated in **Figure** **6**a–c demonstrate fluctuations in response and resistance, respectively, when the BCO‐xLa gas sensors are exposed to different concentrations of nonanal gas (3, 6, 9, 12, 15, 18, 21, 24, 27, and 30 ppm). The sensing for each gas concentration was measured for 6 and 12 min in the presence and absence of gas. Upon exposure to nonanal gas as an electron donor, the electron concentration within BCO‐xLa increased, resulting in a decrease in resistance (Figure 6c). Consequently, the gas sensing responses of the optimal BCO‐6La sensor exhibited a progression from a low level (4.9 at 3 ppm) to a high level (174.6 at 30 ppm) with relatively wide detection range. The measured minimum detection limit of the BCO‐6La sensor (4.9 at 3 ppm) effectively enhances the sensitivity and accuracy of sensors, thereby improving reliability and efficiency in practical applications, particularly in scenarios where high precision and rapid response are required. All the tested sensors responses demonstrate good linearity with the nonanal gas, as shown in Figure 6d. Furthermore, the limit of detection (LOD) for BCO‐6La is estimated to be 117.3 ppb for nonanal, as detailed in the Supporting Information (Note S1, Supporting Information).

**Figure 7:**
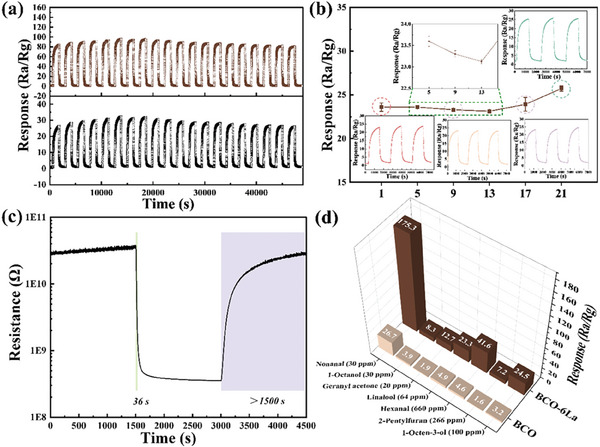
a) Repeatability of the BCO‐6La sensor under 9 and 18 ppm nonanal (20 cycles), b) long‐term stability of the BCO‐6La gas sensor to 9 ppm nonanal, c) responses/recovery time of BCO‐6La to 18 ppm nonanal and d) exclusive detection to 30 ppm nonanal and various gases of BCO‐6La at room temperature (25 ± 2 °C).

For real‐time gas monitoring applications, stable performance is crucial to ensure reliable measurements over extended periods. The BCO‐6La sensor demonstrated highly reproducible sensing characteristics even after undergoing twenty repetitive cycles, as illustrated in **Figure** **7**a. Additionally, the BCO‐6La sensor performance was evaluated over 3 weeks period for 9 ppm nonanal exposure, as shown in Figure 7b. The sensor's response remains stable with variations ≤ 5% (standard deviation), which can be attributed to minor environmental fluctuations (such as humidity or temperature) within the gas mixing setup. Notably, the absence of a decrease in sensor performance emphasizes the reversibility of nonanal interaction. The response and recovery times of BCO‐6La to 18 ppm nonanal gas were determined, as shown in Figure 7c. The response time for nonanal gas was 36 s, while the recovery time exceeded 1500 s, highlighting rapid response capabilities and strong adsorption energy toward nonanal molecules. The swift response time is adequate for periodic nonanal monitoring in critical environments. Furthermore, selectivity plays a crucial role in assessing the performance of gas sensors.^[^ ^51^ ^]^ Therefore, the detection capability of BCO and BCO‐6La sensors was validated by testing their performance against various VOCs that significantly contribute to the flavor of cooked rice, as illustrated in Figure 7d. The BCO‐6La sensor demonstrated enhanced responsiveness to nonanal gas compared to other interfering gases, highlighting its improved selective sensitivity compared to the BCO. This enhanced selectivity may be attributed to the relatively low bond energy of C─H─O in nonanal (≈312.8 kJ mol^−1^), making it susceptible to bond cleavage when reacting with oxygen species.^[^ ^52^, ^53^ ^]^

### 3.3 Gas Sensing Mechanism

When BCO‐xLa is exposed to air, oxygen molecules adsorb on its surface, capturing electrons and forming oxygen ions. At temperatures below 150 °C, oxygen ions predominantly exist as O_2_^−^. The reaction processes are as follows (Equations (2) and (3)):^[^ ^54^, ^55^ ^]^

(2) $$O_{2}\left(\mathrm{gas}\right)\to O_{2}\;(\mathrm{ads})$$

(3) $$O_{2}(\mathrm{ads})\;+\;e^{-}\to {O_{2}}^{-}\;(\mathrm{ads})$$

Upon exposure to nonanal gas, a reducing agent, the nonanal molecules transfer electrons to the conduction band of La‐decorated BCO, reducing the electron depletion layers and potential energy barrier. The reaction processes follow Equations (4) and (5).^[^ ^10^, ^56^, ^57^ ^]^ The resistance of the BCO‐xLa gas sensor decreases, and C_9_H_18_O combines with O_2_^−^ to form C_9_H_16_O, ultimately generating carbon dioxide and water molecules,^[^ ^11^, ^14^ ^]^ which is shown in Figure S6 (Supporting Information).

(4) $$C_{9}H_{18}O(g)\to C_{9}H_{18}O\;(\mathrm{ads})$$

(5) $$2C_{9}H_{18}O(\mathrm{ads})\;+\;{O_{2}}^{-}\left(\mathrm{ads}\right)\to 2C_{9}H_{16}O(\mathrm{ads})\;+\;2H_{2}O(g)+\;e^{-}$$

To further verify the mechanism, we performed in situ FT‐IR spectra of BCO‐6La as shown in Figure S7 (Supporting Information). During the adsorption process of nonanal on BCO‐6La, a distinct strong and broad band ≈2350 cm⁻¹ was observed, attributed to the C═O stretching vibration in carbon dioxide.^[^ ^58^, ^59^ ^]^ Additionally, the broad absorption band at 3389 cm⁻¹ corresponds to the O─H stretching vibration absorption peak of water molecules.^[^ ^60^ ^]^ These results confirm the capability of high‐performance nonanal detection at room temperature.

By introducing O_V_ into the BCO, the oxygen ion conductivity of the materials was enhanced, thereby accelerating the rate of oxidation‐reduction reactions during nonanal detection processes. The enhancement in oxygen ion conductivity can effectively boost the sensitivity and response speed of gas sensors, enabling faster and more accurate gas detection, as corroborated by the gas sensing results in part 3.2. The response of the sensor can be presented as Equation (6):^[^ ^37^ ^]^

(6) $$\;S_{g}=\frac{\Gamma_{t}\;k\left(T\right){\left[O_{\mathrm{ads}}^{\mathrm{ion}}\right]}^{b}}{n_{o}}\;C_{g}^{b}+1$$

where Γ_t_ represents the time constant, k(T) is the reaction rate, ${\left[O_{\mathrm{ads}}^{\mathrm{ion}}\right]}^{b}$ is the density of adsorbed oxygen ions, n_o_ is the electron concentration of the sensor at room temperature, C_g_ is the aimed gas concentration and b is a charge parameter. Specifically, La decoration enhances the ability of the sensor surface to adsorb oxygen (Figure 4a). As a result, there is a significant improvement in the sensor response after La decoration. Notably, the binding energy shift observed between the two components used to reproduce the La 3d_5/2_ peak in Figure 4b (≈3.9 eV) suggests the presence of a small amount of La(OH)_3_. While a minor presence of La(OH)_3_ contributes to improving the sensor response, an excessive amount may be detrimental, as it could impede carrier migration by surrounding the grain boundaries of BCO. As shown in Figure 6, the sensor response gradually decreases with increasing La content. Thus, an optimal level of decoration is beneficial for enhancing gas sensing characteristics, whereas excessive decoration can deteriorate those characteristics. Additionally, as indicated by Equation (6), an increase in the b value correlates with a more rapid increase in response, highlighting the critical role of oxygen adsorption in enhancing gas response. As shown in Figure 6d, the slope b, which relates the sensor sensitivity and target gas concentration, is measured at 0.96, 1.22, 3.44, 6.58, 4.96, and 1.53, respectively for the BCO, BCO‐2La, BCO‐4La, BCO‐6La, BCO‐8La, and BCO‐10La sensors. The slope value of the BCO‐6La sensor is greater than that of BCO, suggesting that the surface of the La‐decorated BCO sensor contains more oxygen ions, particularly in the BCO‐6La samples, which also explains its highest response value.

Furthermore, to achieve a more comprehensive understanding of the gas sensing mechanism of La‐decorated BCO materials, we performed DFT calculations to investigate the role of La as a guest component, as detailed in the Supporting Information (Note S3, Supporting Information). We optimized the adsorption configurations of BCO and BCO‐La. The calculated adsorption energy of nonanal on BCO‐La was found to be −1.56 eV, which is significantly higher than the −0.23 eV observed for BCO (Figure S8, Supporting Information). This indicates that the introduction of La atoms enhances the adsorption of nonanal and subsequent catalytic oxidation, further confirming the strong interaction between BCO‐La and nonanal, elucidating the excellent selectivity of BCO‐La toward nonanal.

### 3.4 Practical Detection Scenario

Monitoring changes in nonanal gas concentration presents a non‐invasive and efficient method for evaluating the quality of cooked rice. For this study, Wuchang rice prepared according to the GB/T 19 266 standard, using a standard domestic rice cooker under specific stages,^[^ ^61^ ^]^ was monitored at room temperature at two time points: fresh and after 6 h of storage. The presence of nonanal in cooked rice has been demonstrated using the gas chromatography‐mass spectrometry (GC‐MS) technique, as shown in Supporting Information (Note S4 and Figure S9, Supporting Information). The BCO‐6La gas sensor was used to monitor electrical resistance changes during rice storage (Note S5 and Figure S10, Supporting Information). To minimize the influence of humidity, calcium chloride (CaCl_2_) was used as a desiccant without affecting nonanal detection. Results demonstrated a significant increase in the sensor response after 6 h of rice storage compared to the fresh state, indicating a rise in nonanal concentration. This observation highlights the potential of the sensor to detect changes in nonanal concentration, reflecting the progression of staling processes in cooked rice. These findings are consistent with previous studies, which suggest that minimizing the oxidation and hydrolysis of lipids, and moisture loss during storage can preserve the freshness of cooked rice.^[^ ^62^, ^63^, ^64^ ^]^

## 4 Conclusion

In this study, La‐decored BCO microspheres characterized by an abundance of oxygen vacancies (O_V_) were synthesized. The gas sensor engineered using BCO‐6La microspheres as active layer demonstrated exceptional sensitivity for room temperature detection of nonanal at a concentration of 30 ppm. Notably, the sensor exhibited a rapid response time of 36 s, excellent response of 174.6, and good selectivity that was approximately 4 to 24 times greater than that for other target gases. The enhanced sensing performance was attributed to the engineered O_V_ in the BCO‐6La microspheres, which effectively reduces the energy bandgap, thereby facilitating electron transfer enhancing the sensor response to nonanal. Therefore, the incorporation of extrinsic oxygen defects through the La decoration of BCO microspheres is crucial for optimizing the nonanal sensing performance. We anticipate that this research will contribute significantly to advancements in nonanal gas sensors based on BCO.

## Conflict of Interest

The authors declare no conflict of interest.

## Supporting information



Supporting Information

## Data Availability

The data that support the findings of this study are available from the corresponding author upon reasonable request.
