# Supplementary material for: Decorated‐Induced Oxygen Vacancy Engineering for Ultra‐Low Concentration Nonanal Sensing: A Case Study of La‐Decorated Bi2O2CO3
Source: Adv Sci (Weinh). 2024 Sep 28;11(43):2408096. doi: 10.1002/advs.202408096 (PMC11578350; doi:10.1002/advs.202408096)
Supplement: Supplementary file 1 — Supporting Information [file ADVS-11-2408096-s001.docx]

**Supplementary information for**

**Decorated‐Induced Oxygen Vacancy Engineering for Ultra-low Concentration Nonanal Sensing: A Case Study of La-Decorated Bi_2_O_2_CO_3_**

Zichen Zheng^a,b^, Kewei Liu^a,c^, Yiwen Zhou^a^, Kaichun Xu^a^, Yifan Luo^a^, Jiabao Ding^a^, Carla Bittencourt^b^, Marc Debliquy^c^, Chao Zhang^a,^*

*a. College of Mechanical Engineering, Yangzhou University, Yangzhou 225127, PR China*

*b. Research Institute for Materials Science and Engineering, Chimie des Interactions Plasma‐Surface, University of Mons, 20 Place du Parc, 7000 Mons, Belgium*

*c. Service de Science des Matériaux, Faculté Polytechnique, University of Mons, 7000 Mons, Belgium*

*Corresponding authors.

E-mail addresses: zhangc@yzu.edu.cn (Chao Zhang)

**Table S1** XRD parameters of BCO.

| Peak | 2 (°) | β (°) | D (nm) | ×10^-3^ (nm^-2^) | ε×10^-3^ |
| --- | --- | --- | --- | --- | --- |
| 1 | 12.69023 | 1.44401 | 5.535455953 | 32.63572022 | 56.66177971 |
| 2 | 24.02729 | 0.7192 | 11.29335447 | 7.84068634 | 14.74633987 |
| 3 | 30.07053 | 1.42166 | 5.786117186 | 29.86933473 | 23.09365054 |
| 4 | 32.73249 | 0.43114 | 19.20439923 | 2.711430948 | 6.405710598 |
| 5 | 35.14151 | 0.45604 | 18.27267206 | 2.994993471 | 6.28392844 |
| 6 | 42.32227 | 1.29602 | 6.572981512 | 23.14595895 | 14.60872581 |
| 7 | 46.92176 | 0.66093 | 13.10306731 | 5.824438398 | 6.644806747 |
| 8 | 48.64232 | 1.06312 | 8.200392049 | 14.87067795 | 10.26355355 |
| 9 | 51.99659 | 1.62754 | 5.43071328 | 33.9067568 | 14.56129747 |
| 10 | 53.38077 | 0.88625 | 10.03298356 | 9.934357833 | 7.69187939 |
| 11 | 56.76314 | 1.10159 | 8.196913333 | 14.88330266 | 8.896446676 |

In Table S1-S6, 2 is the peak position, and β is it’s the corresponding FWHM. From XRD data, D (nm) is the crystallite size calculated through the Scherer equation ($D=\frac{k}{cos}$). (nm^-2^) is the dislocation density calculated through $=\frac{1}{D^{2}}$ equation. ε is the microstrain calculated by $\varepsilon=\frac{\beta}{4 tan}$.

**Table S2** XRD parameters of BCO-2La.

| Peak | 2 (°) | β (°) | D (nm) | ×10^-3^ (nm^-2^) | ε×10^-3^ |
| --- | --- | --- | --- | --- | --- |
| 1 | 12.65575 | 1.03541 | 7.719634421 | 16.78056317 | 40.74024737 |
| 2 | 23.99391 | 0.79293 | 10.24261589 | 9.531872548 | 16.28138009 |
| 3 | 30.02862 | 1.39182 | 5.909588798 | 28.63422772 | 22.64197531 |
| 4 | 32.69429 | 0.4213 | 19.65101979 | 2.589582872 | 6.26723858 |
| 5 | 35.05813 | 0.51904 | 16.05107892 | 3.881428046 | 7.170150378 |
| 6 | 42.28197 | 1.36942 | 6.219827731 | 25.84897267 | 15.45223004 |
| 7 | 46.87816 | 0.65501 | 13.21931128 | 5.722454412 | 6.592155376 |
| 8 | 48.39075 | 1.41252 | 6.165845557 | 26.30357117 | 13.71688383 |
| 9 | 51.98088 | 1.47633 | 5.986542473 | 27.90280499 | 13.21304718 |
| 10 | 53.32824 | 0.89423 | 9.941160431 | 10.11872598 | 7.770012412 |
| 11 | 56.73578 | 1.06525 | 8.47545023 | 13.92112856 | 8.607878397 |

**Table S3** XRD parameters of BCO-4La.

| Peak | 2 (°) | β (°) | D (nm) | ×10^-3^ (nm^-2^) | ε×10^-3^ |
| --- | --- | --- | --- | --- | --- |
| 1 | 12.66555 | 1.15146 | 6.941676228 | 20.75254159 | 45.27112057 |
| 2 | 24.0309 | 0.76278 | 10.6482013 | 8.819571684 | 15.63747611 |
| 3 | 30.07621 | 1.4251 | 5.772227136 | 30.01326036 | 23.14495116 |
| 4 | 32.7378 | 0.43396 | 19.07986305 | 2.746942026 | 6.44650414 |
| 5 | 35.06466 | 0.66998 | 12.43515026 | 6.466926499 | 9.253437834 |
| 6 | 42.3154 | 1.40955 | 6.043430758 | 27.37996564 | 15.89126487 |
| 7 | 46.91862 | 0.60109 | 14.40733882 | 4.817619111 | 6.043645446 |
| 8 | 48.81045 | 0.45446 | 19.19595814 | 2.71381608 | 4.370342439 |
| 9 | 51.9963 | 1.58584 | 5.573508161 | 32.19161145 | 14.18830647 |
| 10 | 53.34656 | 0.90427 | 9.831574357 | 10.34555666 | 7.854119223 |
| 11 | 56.7736 | 1.03744 | 8.704197964 | 13.19904411 | 8.376542519 |

**Table S4** XRD parameters of BCO-6La.

| Peak | 2 (°) | β (°) | D (nm) | ×10^-3^ (nm^-2^) | ε×10^-3^ |
| --- | --- | --- | --- | --- | --- |
| 1 | 12.56939 | 1.41952 | 5.630298272 | 31.54548395 | 56.24069263 |
| 2 | 24.07105 | 0.78365 | 10.36539429 | 9.307399304 | 16.03772504 |
| 3 | 30.11184 | 1.41764 | 5.803087178 | 29.69489611 | 22.99525744 |
| 4 | 32.77612 | 0.43908 | 18.8592311 | 2.811590292 | 6.514504692 |
| 5 | 35.146 | 0.61979 | 13.44515524 | 5.531824048 | 8.539132146 |
| 6 | 42.37022 | 1.26365 | 6.742449494 | 21.99705781 | 14.22616603 |
| 7 | 46.95964 | 0.62035 | 13.96220419 | 5.129700744 | 6.231185814 |
| 8 | 48.83795 | 0.40817 | 21.37527355 | 2.188651618 | 3.92268966 |
| 9 | 52.02679 | 1.52305 | 5.804037674 | 29.68517096 | 13.61733485 |
| 10 | 53.39904 | 0.90116 | 9.867775487 | 10.26978805 | 7.818178894 |
| 11 | 56.80025 | 1.06366 | 8.490700419 | 13.87116585 | 8.583475851 |

**Table S5** XRD parameters of BCO-8La.

| Peak | 2 (°) | β (°) | D (nm) | ×10^-3^ (nm^-2^) | ε×10^-3^ |
| --- | --- | --- | --- | --- | --- |
| 1 | 24.34566 | 1.09432 | 7.426545155 | 18.13119163 | 22.13547036 |
| 2 | 29.33996 | 2.48899 | 3.299328375 | 91.86475391 | 41.48542167 |
| 3 | 32.70133 | 0.51104 | 16.20053977 | 3.810140853 | 7.600477576 |
| 4 | 42.71274 | 2.01033 | 4.243092563 | 55.54372321 | 22.43305869 |
| 5 | 46.88054 | 0.89147 | 9.713012275 | 10.59966464 | 8.971426886 |
| 6 | 53.36292 | 1.26441 | 7.031766209 | 20.22419081 | 10.97824032 |
| 7 | 56.4755 | 1.53065 | 5.891246797 | 28.81280677 | 12.43607972 |

**Table S6** XRD parameters of BCO-10La.

| Peak | 2 (°) | β (°) | D (nm) | ×10^-3^ (nm^-2^) | ε×10^-3^ |
| --- | --- | --- | --- | --- | --- |
| 1 | 29.14384 | 5.07344 | 1.617902196 | 382.0282431 | 85.1565443 |
| 2 | 31.30176 | 1.53261 | 5.383090099 | 34.50934388 | 23.86926359 |
| 3 | 46.93435 | 0.97755 | 8.859519504 | 12.74030024 | 9.825060463 |
| 4 | 53.48595 | 2.4501 | 3.630807061 | 75.85664962 | 21.21618975 |
| 5 | 56.55071 | 2.68359 | 3.361399628 | 88.50334897 | 21.76908749 |


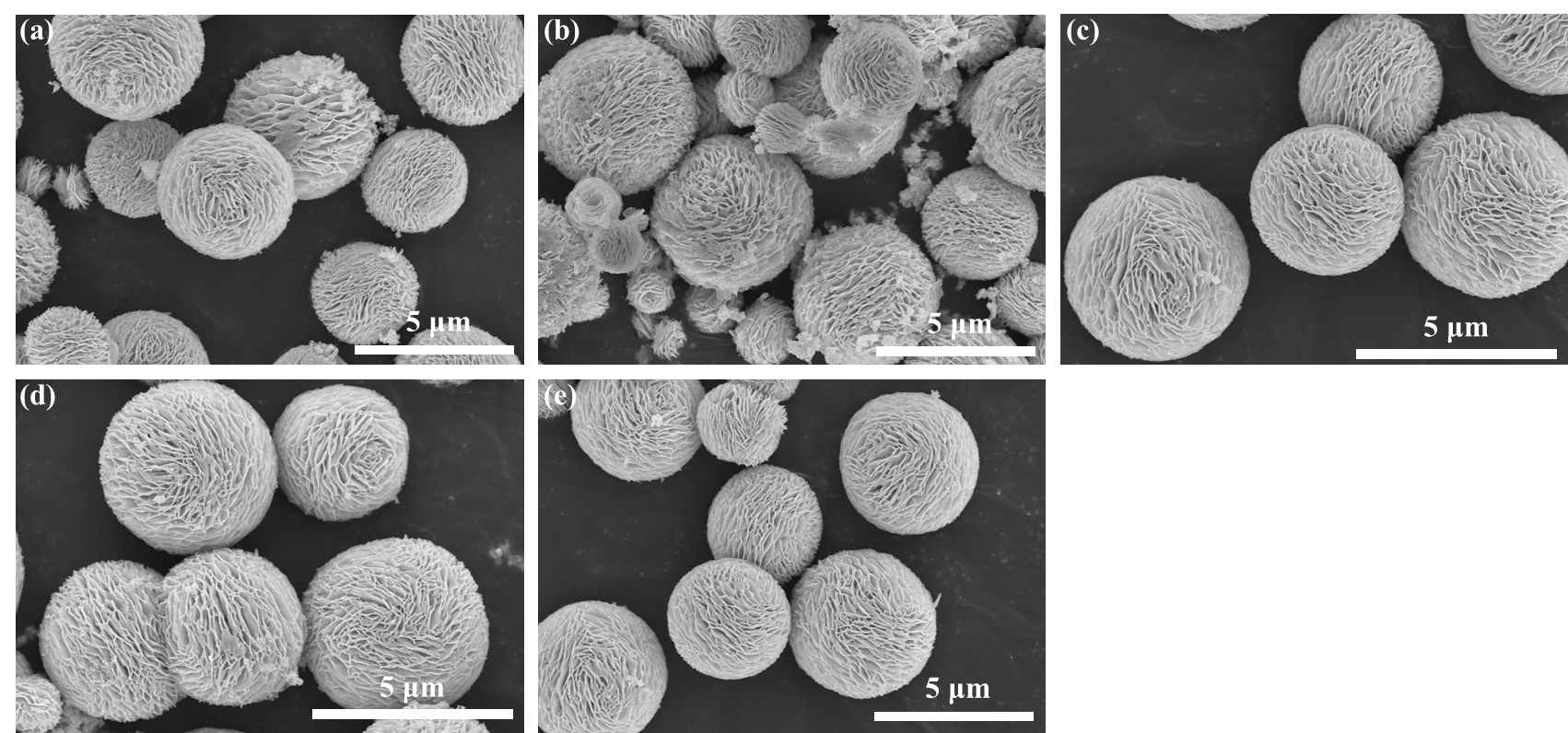


**Fig. S1** FESEM image of (a) BCO, (b) BCO-2La, (c) BCO-4La, (d) BCO-8La and (e) BCO-10La.


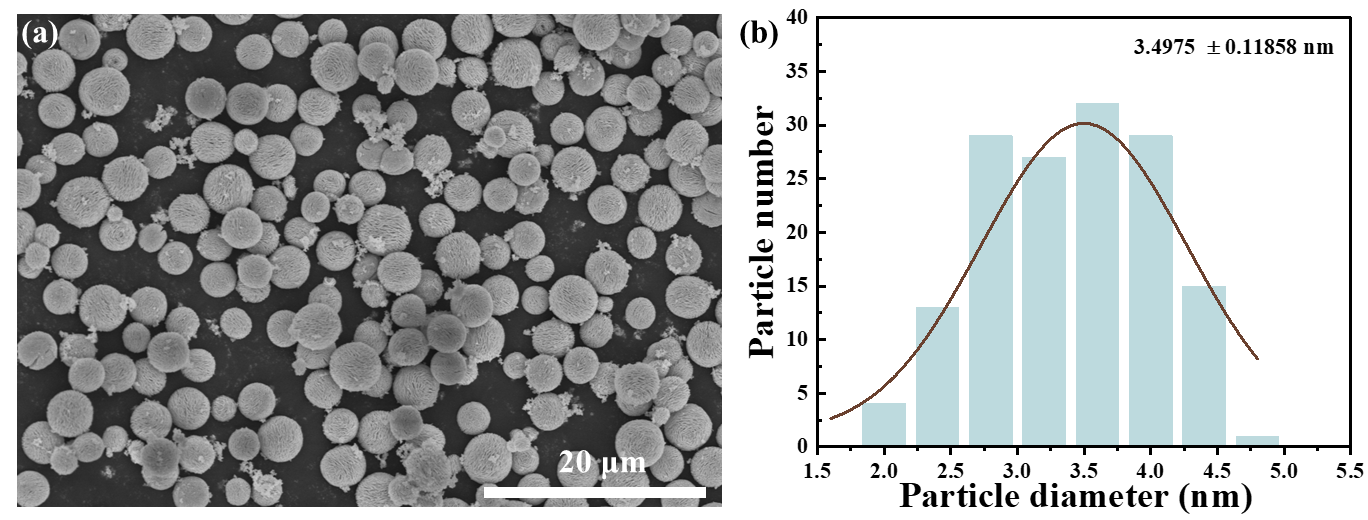
**Fig. S2** FESEM image of (a) BCO-6La, (b) the particle size distribution of BCO-6La.


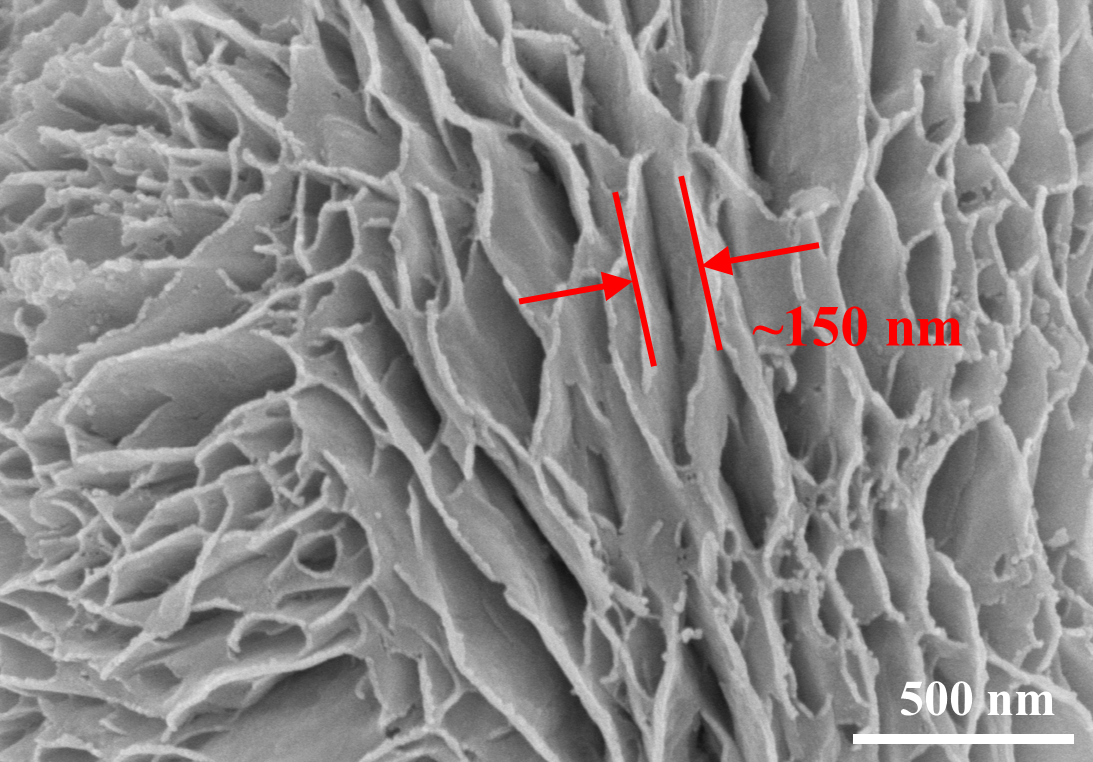


**Fig. S3** FESEM image of BCO-6La.


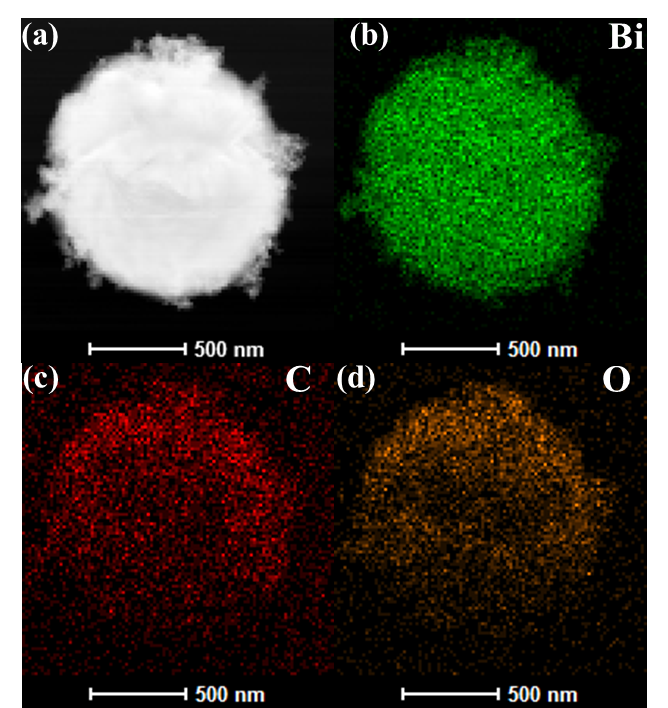


**Fig. S4** (a-d) EDS elemental mapping results of BCO.

**Fig. S5** XPS survey spectrum of BCO and BCO-6La.

**Note 1 Limit of detection (LOD) calculation**

The signal is valid if the ratio of signal-to-noise is equivalent to 3. Therefore, the theoretical LOD could be calculated as:

$LOD(ppm)=3\frac{\sigma}{k}$ (S1)

$\sigma=\sqrt{\frac{\sum{(y-y_{i})}^{2}}{N}}$ (S2)

where k is the slop of the linear calibration curve of response value vs. gas concentration (ppm), $\sigma$ is the sensor noise. y, y_i_, N are the baseline data point (100), the average, and the number of data points, respectively. All the base line data can be obtained from Fig. 6.

**Note 2 Selectivity detection**

All the liquid VOCs were purchased from Shanghai Aladdin Biochemical Technology Co., Ltd., the vapor pressures of 1-octen-3-ol, geranyl acetone, 1-octanol, hexanal, 2-pentyl-furan, and linalool are 0.75, 0.0157, 0.14, 10, 2.02 and 0.17 mmHg at room temperature, respectively. The concentrations of VOC gases were calculated through Equation (S3) ^1^. The flow rates of dry air (y, in sccm) are derived from high-purity mixed air sources, and were regulated using two separate mass flow controllers. The concentration of selected interfering VOC (in ppm) was calculated as 100x/(x+y), where the flow of each component was individually adjusted.

$\text{Concentration of VOCs (ppm)=(}\text{vapor pressure of VOCs(mmHg)/760)×}\text{10}^{\text{6}}$ (S3)


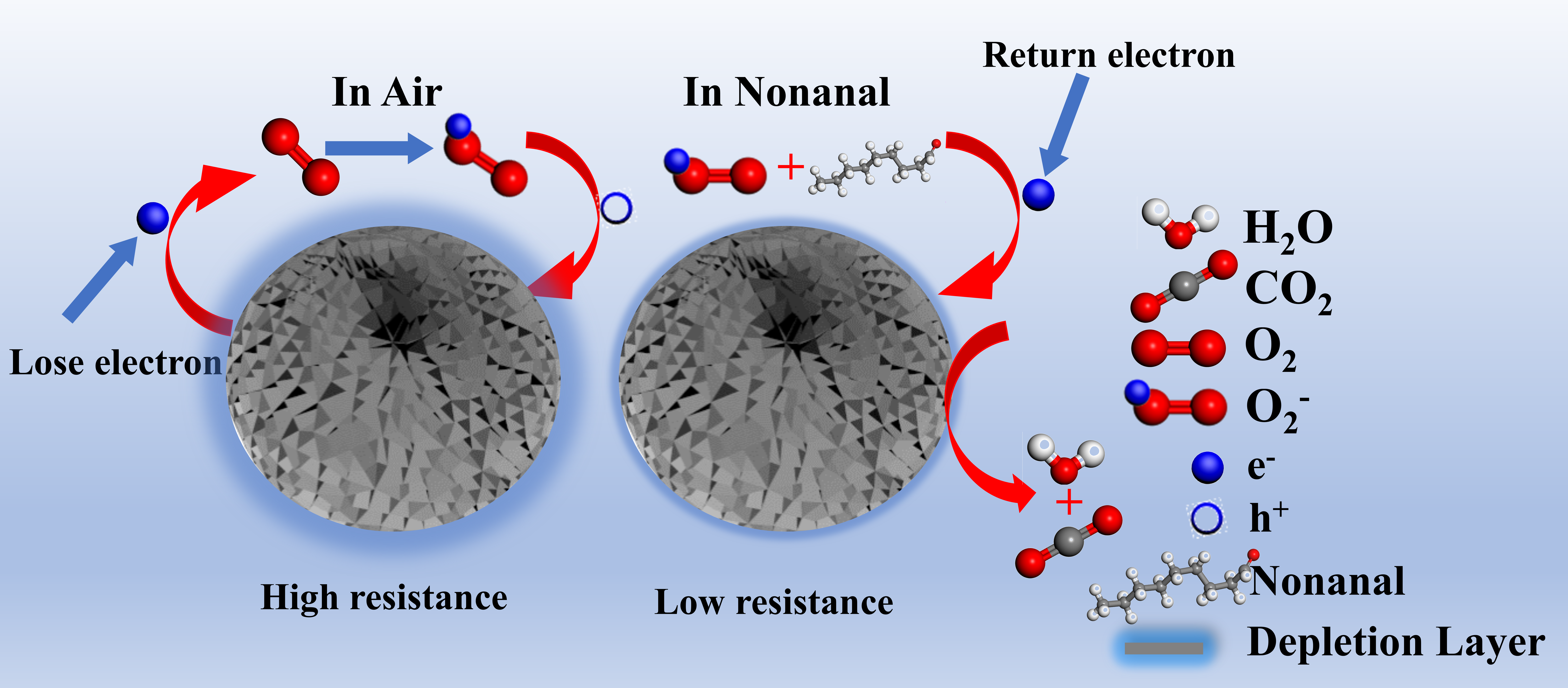


**Fig. S6** Schematic illustration of the nonanal gas-sensing mechanism of La doped Bi_2_O_2_CO_3_ microspheres at room temperature.


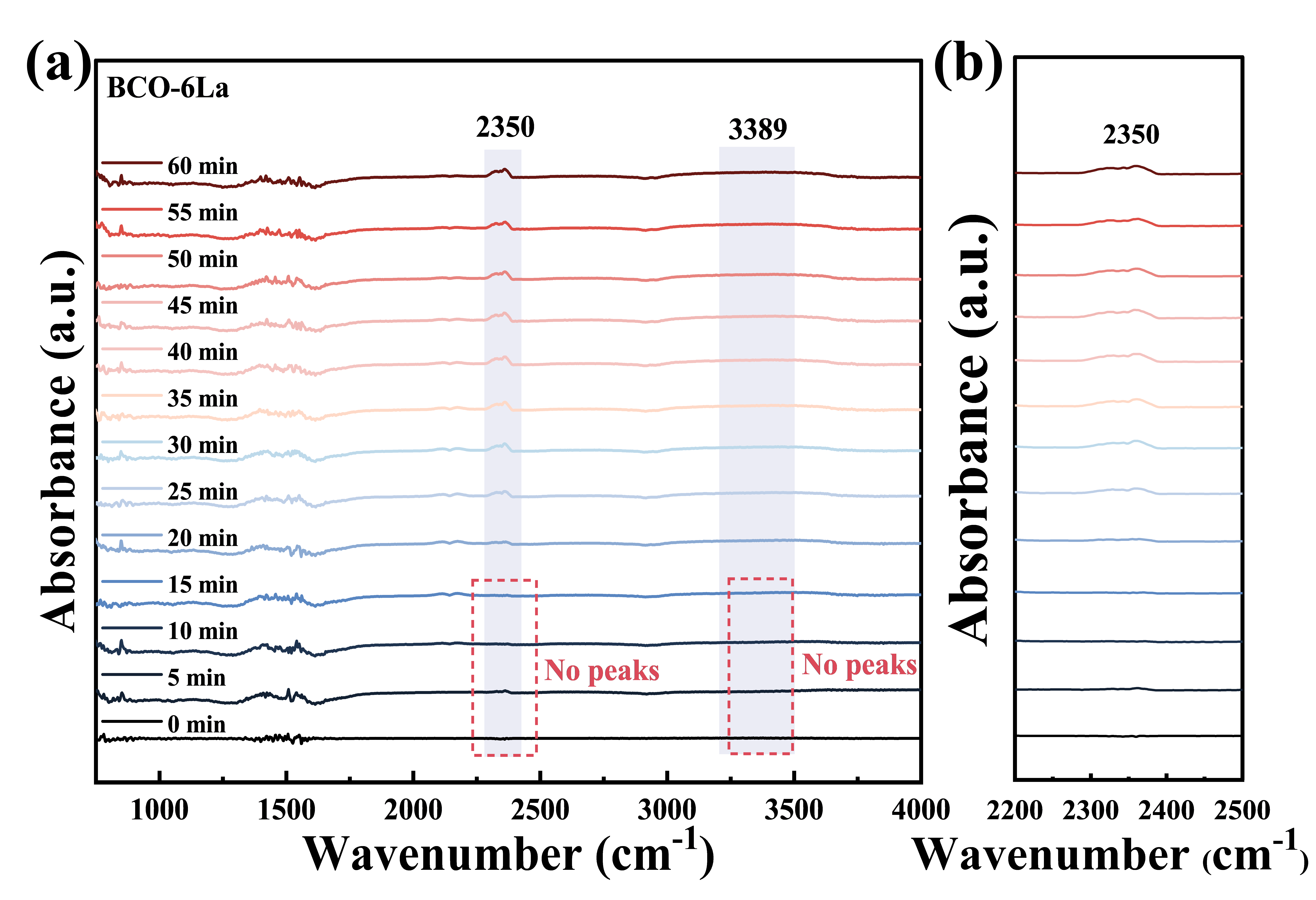


**Fig. S7** (a) In-situ FT-IR spectra of BCO-6La during nonanal adsorption, (b) magnified portion of In-situ FT-IR spectra (2200 cm^-1^-2500 cm^-1^).

**Note 3**

**DFT calculation details**

Decoration configurations and the nonanal molecular adsorption behavior were revealed by DFT calculations. All DFT analysis was calculated by CASTEP code of materials studio. In the generalized gradient approximation (GGA), the exchange-related potential was described by the Perdew-Burke-Ernzerhof (PBE) functional. The 2×2×1 supercells of the (020) surface Bi_2_O_2_CO_3_ were built. When performing geometric optimization, the energy cutoff of plane wave expansion was set to 500 eV, and the energy convergence criterion in geometry optimization was set as 10^-5^ eV. A 15 Å vacuum layer was set between the layers to avoid interactions. The adsorption energy was calculated by equation (S4):

$E_{\mathrm{ads}}$=$E_{surface+gas}-(E_{\mathrm{surface}}+E_{\mathrm{gas}})$ (S4)

where E_surface_, E_gas_, and E_surface+gas_ denote the total electronic energies of BCO/BCO-La, nonanal, and their complex, respectively.

**
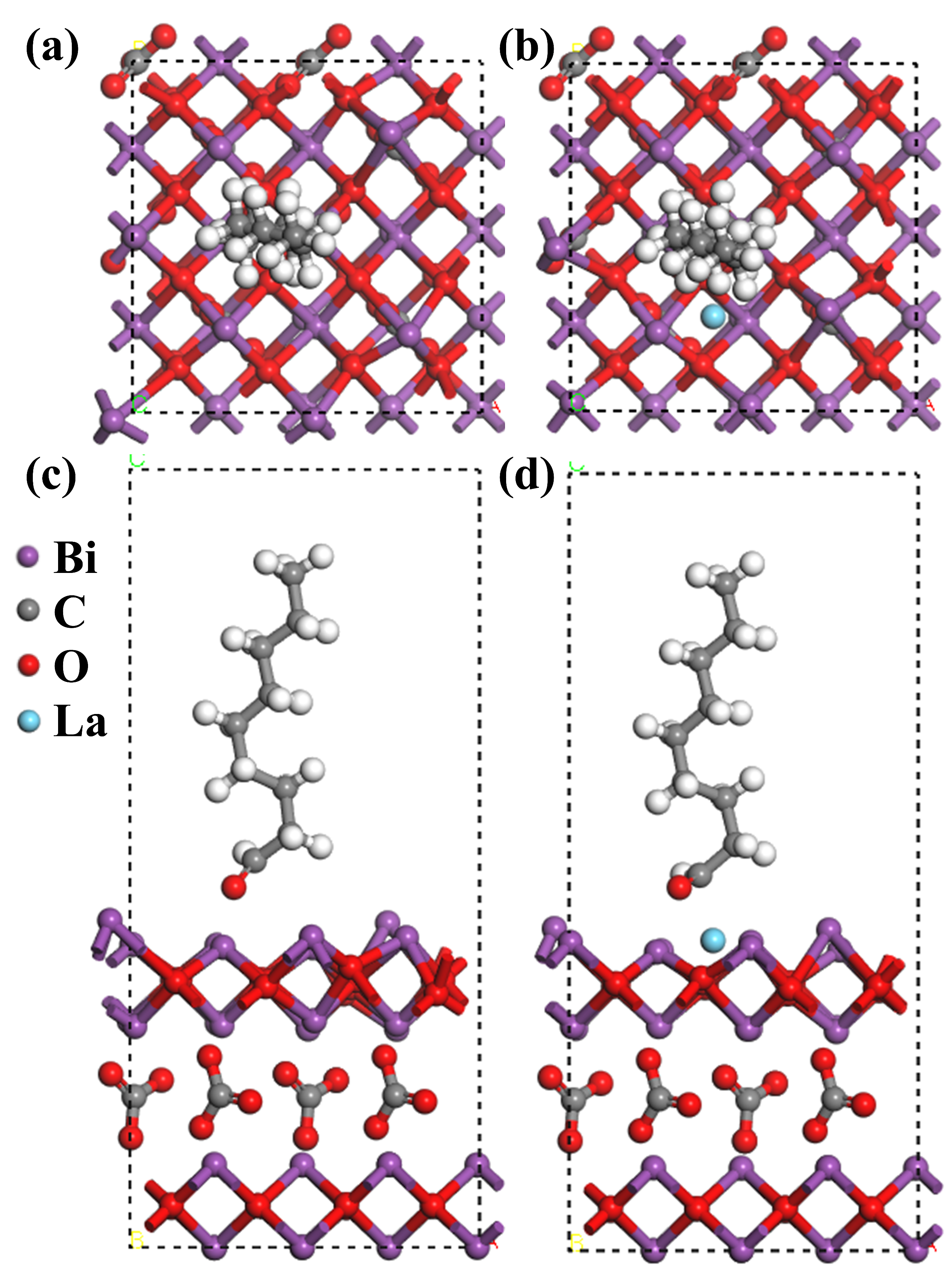
**

**Fig. S8** DFT calculation of the nonanal molecule adsorption energies of BCO and BCO-6La. (a-b) Optimized adsorption structures of nonanal on the BCO and BCO-6La in the top view, respectively. (c-d) Adsorption configurations and energies of nonanal on the optimum structure of BCO and BCO-6La, respectively.

**Note 4**

**Volatile compounds analysis**

Volatile compounds were extracted by headspace solid-phase microextraction (HS-SPME) and analyzed by gas chromatography coupled with a mass spectrometer (Trace ISQ, Thermofisher, USA). The SPME device contained fused-silica fiber (Supelco, Bellefonte, PA, USA) coated with DVB/CAR/PDMS (Divinylbenzene/Carboxen/Polydimethylsiloxane, 50/30 μm thickness with a 10 mm length). The cooked rice samples were weighed (20 g) and placed into 20 mL headspace vials (sealed with a PTFE silicone septum). The vials were maintained at 50°C for 60 min to equilibrate the absorption of the volatile compounds by SPME fiber. Then, the fiber was desorbed at the GC-MS injection port for 5 min at 250°C. The volatiles were separated by DB-5MS capillary column (30 m × 0.25 mm × 0.25 μm) at a column velocity of 6 PSI. The injection port was in a split-less mode. The temperature program was firstly set at 40°C for 5 min, raised to 125°C at a rate of 8°C/min, and kept for 3 min, then raised to 165°C at a rate of 4°C/min, and kept for 3 min, then raised to 250°C at a rate of 3°C/min, and kept for 2 min. The MS detector conditions were as follows: mass spectra were operated by electron ionization mode with 70 eV electron energy, ion source temperature was 250°C, multiplier voltage was 1000 V, and mass spectra range was from 20 to 400 m/z. Compounds were identified by comparing their mass spectra with those contained in the mass spectra libraries (NIST 11, WILEY 07) and by calculating their linear retention index (RI) in relation to standard n-alkanes (C7–C40) and matching them with literature data.

**

**

**Fig. S9** (a) Magnified chromatogram of the nonanal region of cooked rice, (b-c) mass spectra of the nonanal in cooked rice.

**Note 5**

**Methods of verifying practical experiment**

The cooking process follows the four steps: (I) around 25 min from the beginning of heating until steam comes out; (II) the steam starts to come out of the pot and ends 13 min later; (III) the remaining steam overflows from the rice cooker until it stops automatically 10 min of heating; (IV) 30 min of heat preservation starting from automatic stop heating. In detail, 20 g of cooked rice were weighed and sealed in a sample container with a volume of 100 mL. High-purity mixed air (Nanjing Special Gas Factory Co., Ltd.) blew through the sample. The produced VOCs were further diluted before being exposed to the surface of the sensing material. The pump draws volatile organic compounds from the cooked rice into the gas chamber, ensuring smooth airflow.”

**Fig. S10** Response curves of the BCO-6La sensor as a function of storage time measured at room temperature under exposure to odors from cooked rice.

**Reference**

(1) Liu G.; Froudarakis E.; Patel J. M.; Kochukov M. Y.; Pekarek B.; Hunt P. J.; Patel M.; Ung K.; Fu C. H.; Jo J.; Lee H. K.; Tolias A. S.; Arenkiel B. R. Target specific functions of EPL interneurons in olfactory circuits, *Nature Communications* **2019**, 10, 3369.
